# Radiopacity evaluation of calcium silicate cements

**DOI:** 10.1186/s12903-023-03182-w

**Published:** 2023-07-15

**Authors:** Havva Gozde Sen, Dilek Helvacioglu-Yigit, Ayca Yilmaz

**Affiliations:** 1 Department of Endodontics, Faculty of Dentistry, Istanbul University, Istanbul, Turkey; 2 Institute of Health Sciences, Istanbul University, Istanbul, Turkey; 3 College of Dental Medicine, QU Health, Qatar University, P.O. Box:2713, Doha, Qatar

**Keywords:** Biodentine, Calcium silicate cements, NeoMTA 2, OrthoMTA, ProRoot MTA, Radiopacity

## Abstract

**Background:**

The aim of this study was to compare the radiopacity of calcium silicate cements using a digital imaging method.

**Methods:**

Four calcium silicate cements, NeoMTA 2, OrthoMTA, ProRoot MTA, and Biodentine, were used in this study. Disk-shaped samples were prepared from each material and placed on a plexiglass plate. An aluminum step-wedge was placed alongside the samples on a digital sensor and exposed to 70 kVp and 8 mA from 30 cm away for 0.32 s. The greyness values ​​of the tested materials were measured digitally with the system software and compared with those of the step-wedge to determine the equivalent aluminum thickness.

**Results:**

The radiopacity values, expressed in equivalent millimetres of aluminum, of the studied materials ProRoot MTA, OrthoMTA, NeoMTA 2, and Biodentine were 4.32 ± 0.17 mm Al, 3.92 ± 0.09 mm Al, 3.83 ± 0.07 mm Al, and 2.29 ± 0.21 mm Al, respectively. Statistically significant differences were found between the mean radiographic density values of the tested materials (p < 0.05).

**Conclusion:**

ProRoot MTA was the most radiopaque root canal filling material among the tested materials. All materials, except Biodentine, were found to be compliant with the minimum radiopacity requirements of ISO 6876 and ADA 57 standards.

## Background

Radiopacity is an essential property of endodontic materials, which should be more radiopaque than dentin. The International Organization for Standardization (ISO 13116:2014 and ISO 6876:2012) and American National Standards Institute/American Dental Association (ANSI/ADA57:2021) standards are used to determine radiopacity of root canal sealers. The radiopacity of an ideal root canal filling should be sufficient to support its physical and chemical properties [1–3], both standards require more than 3 mm Al for root canal sealers [4] for a 1 mm thick sample.

To determine the radiopacity of a particular material, a disk of specified thickness is made from the material and radiographed and compared to a step-wedge of aluminum. Expressing radiopacity as the equivalent thickness of the material in Al minimizes the potential effects of the exposure time. Digital systems use slightly different methods to measure X-ray radiation, which may be different from those of conventional film radiography. Digital radiography, as clinically relevant digital equipment in dentistry, can influence the measured radiopacity of a material [5].

Calcium silicate cement-based materials are used in many endodontic applications, including perforation repair, pulp capping, apexification, root canal filling, retro-filling, and resorption repair. ProRoot MTA (Dentsply Sirona, Johnson City, TN) was released in 1998 as the first commercial mineral trioxide aggregate (MTA) product. It is mainly composed of calcium silicate cement with 20% bismuth oxide. Its cytotoxicity is low, and it is biocompatible. ProRoot MTA has good sealing properties, is not affected by blood and is effective in a moist environment, making such calcium silicate cement materials desirable [6]. Bismuth oxide, the radiopacifier present in ProRoot MTA, is a compound that causes long-term discolouration [7].

Alternatives to bismuth oxide have been used by manufacturers. Biodentine (Septodont, Saint-Maur-des-Fossés, France) contains zirconium oxide as a radiopacifier [8]. Biodentine contains tricalcium silicate (calcium silicate cement) and zirconium oxide. Biodentine has many favourable properties but has low radiopacity [9]. Biodentine can be used as a temporary restorative material because of its high strength [10].

OrthoMTA (BioMTA, Seoul, Korea) is a calcium silicate cement that was introduced in 2007 and forms a hydroxyapatite layer to prevent microleakage [11]. Ortho MTA contains bismuth oxide as a radiopacifier. NeoMTA 2 (Avalon Biomed, Houston TX, USA) contains tantalite instead of bismuth oxide, allowing it to eliminate discolouration with faster setting than ProRoot MTA while having the same bioactivity as the other materials [12].

In this study, the radiopacity of four calcium silicate-based silicate cement products were compared. The aim of this study was to measure the radiopacity of ProRoot MTA, Biodentine, OrthoMTA, and NeoMTA 2 using digital radiography. The null hypothesis of the present study was that new calcium silicate cement materials, NeoMTA 2 and OrthoMTA, have radiopacity values similar to those of the other calcium silicate cement products.

## Methods

The study was carried out in the Department of Endodontics, Faculty of Dentistry, Istanbul University between March and July 2022. Four calcium silicate cement materials were tested: NeoMTA 2, OrthoMTA, Biodentine, and ProRoot MTA. The compositions and manufacturers of the materials are listed in Table 1.

**Table 1:** Information on the commercial tested materials [13–15]

Material	Composition	Manufacturer recommended mixing ratios	Manufacturer	Lot #
ProRoot MTA	Powder: Tricalcium silicate, dicalcium silicate, tricalcium aluminate, bismuth oxide, and gypsum Liquid: Distilled water	0.5 gr powder and a micro-dose ampoule of liquid	Dentsply Sirona, Johnson City, TN	0000249679
OrthoMTA	Powder: Tricalcium silicate, dicalcium silicate, tricalcium aluminate, tetracalcium aluminoferrite, free calcium oxide, bismuth oxide Liquid: Distilled water	0.2 gr powder and two drops of liquid	BioMTA, Seoul, Korea	OMCA02D05
NeoMTA 2	Powder: Tricalcium silicate, dicalcium silicate, tantalum oxide, and minor amounts of calcium sulfate and tricalcium aluminate Liquid: Water and polymers	One scoop of powder and one or two drops of liquid, according to the desired consistency (putty or sealer)	Avalon Biomed, Houston, TX, USA	2021031603
Biodentine	Powder: Tricalcium silicate, calcium carbonate, zirconium oxide, dicalcium silicate, and minor additives of iron oxide Liquid: Aqueous solution of a hydrosoluble polymer with calcium chloride	One capsule of Biodentine to five drops of liquid	Septodont, Saint-Maur-des-Fossés, France	B26176

Plexiglass moulds with cavities 1 mm in depth and 10 mm in diameter, were fabricated. The calcium silicate cements were mixed according to the manufacturer instructions. NeoMTA 2 was prepared in thin consistency (one scoop of powder and 2 drops of liquid). Each of the mixed calcium silicate cements was placed in the plexiglass cavities. A glass plate was placed on top to keep the thickness uniform. Ten samples were prepared from each material. The samples placed in the plexiglass cavities were kept in an incubator at 37 °C for 24 h in 95% humidity, and they were completely hardened. The samples were removed from the plexiglass cavities and their thicknesses were measured with a digital calliper and inserted back into the cavities.

### Radiographs

**Fig. 1:**
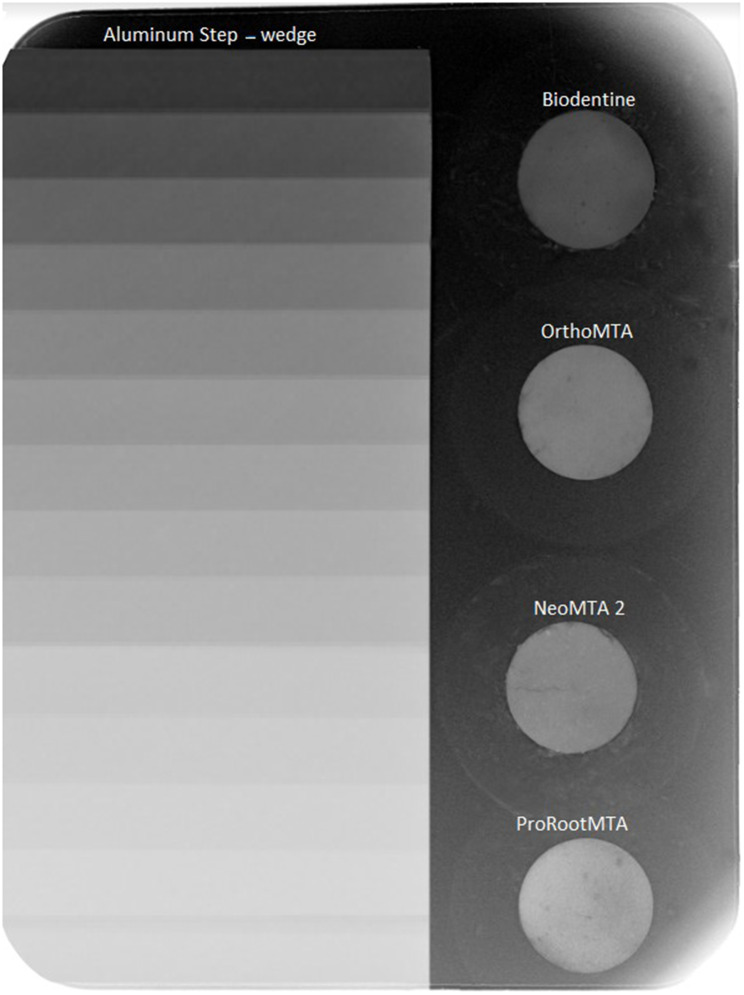
Digital radiographic image of root calcium silicate cement samples and aluminum step-wedge

A 14-step-wedge aluminum plate with a step thickness of 1 mm was fabricated. The chemical composition of the plate was 99.12% Al, 0.47% Fe, 0.41% Mg, and < 0.1%Cu, which conformed to ISO 13,116. The specimens and a step-wedge were radiographed using an intraoral radiography device, X-ray phosphor plate, and image plate scanner (Vistascan Mini, Dürr Dental, Bietigheim-Bissingen, Germany). The obtained intraoral radiography device was set to 70 kVp and 8 mA. The focal spot and object distance was set to 30 cm, and the exposure time was 0.32 s (Fig. 1). The X-ray plate was immediately scanned after exposure.

**Fig. 2:**
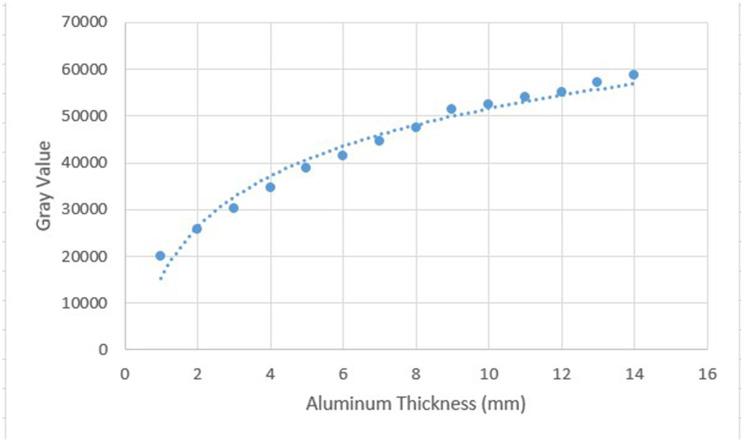
Graph showing mean grey values vs. equivalent aluminum thickness (mm Al). The curve was created using the Curve Expert Professional software to calculate the aluminum thickness equivalency for all tested materials

Digital radiographs (Vistascan Mini, Dürr Dental, Bietigheim-Bissingen, Germany) were input into Mediadent (Morita-IC5-HD software), and the average greyness of each step and the selected material was determined using this software. The measurement was carried out by an operator who was not informed about the identity of the materials. Air bubbles were avoided. The levels were determined at which the average greyness values of the calcium silicate cements were equal to that of the aluminum step-wedge. This procedure was repeated five times for each of ten samples of the material with the aluminum step-wedge, and then the average values were calculated. A graph correlating the Al thickness to the mean grey values was created (Fig. 2). The average grey value of each material was converted into the equivalent aluminum thickness (mm Al) using Curve Expert Professional software (www.curveexpert.net).

### Statistical methods

The data were analysed using IBM SPSS version 23. Conformity to the normal distribution was evaluated using the Shapiro–Wilk test. The radiopacity values of the materials were compared with a one-way analysis of variance, and multiple comparisons were analysed with Tukey’s honestly significant difference test (Tukey’s HSD test). The results of the study are presented as the means ± standard deviations. The significance level was p < 0.050.

## Results

Differences were determined among the mean radiopacity values of the materials (p < 0.001). All the cements except for Biodentine exceeded the ISO 6876 requirement of 3 mm Al. As shown in Table 2, no statistically significant difference were determined between the mean radiopacity values of NeoMTA 2 and OrthoMTA (p = 0.157). A statistically significant difference was observed between the mean radiopacity values of NeoMTA 2 and Biodentine (p < 0.001), NeoMTA 2 and ProRoot MTA (p < 0.001), and OrthoMTA and Biodentine (p < 0.001). A statistically significant difference was also observed between the mean radiopacity values of OrthoMTA and ProRoot MTA (p = 0.001) and Biodentine and ProRoot MTA (p < 0.001). ProRoot MTA showed a significantly higher radiopacity than OrthoMTA, NeoMTA2, and Biodentine. Both OrthoMTA and NeoMTA 2 showed significantly higher radiopacity than Biodentine.

**Table 2:** Millimetre aluminum equivalents of the radiopacity values of the calcium silicate cements

Calcium silicate cements	Radiopacity mm Al (Mean ± SD)
NeoMTA 2	3.83 ± 0.07^a^
OrthoMTA	3.92 ± 0.09^a^
Biodentine	2.29 ± 0.21^b^
ProRoot MTA	4.32 ± 0.17^c^

One-way analysis of variance (ANOVA), post hoc Tukey’s HSD test

Different superscript small letters represent significant differences (p < **0.001**)

## Discussion

The aim of this study was to compare the radiopacity of calcium silicate cement-based materials by digital radiography. This study compared the radiopacity properties of NeoMTA 2, OrthoMTA, ProRoot MTA and Biodentine. The null hypothesis of the present study was rejected since NeoMTA 2 and OrthoMTA showed significantly lower radiopacity than ProRoot MTA, while both had higher radiopacity than Biodentine.

The molecular weight and thickness of a material determine its radiopacity [16, 17]. Eliasson and Haasken [18] were the first researchers to establish an equivalent aluminum thickness using optical radiographic density values as a standard of comparison of materials. Beyer-Olsen and Ørstavik [19] modified this model; they measured the amount of light transmitted through an X-ray film with an optical densitometer. They converted the light transmission into an equivalent aluminum thickness by comparing it with an aluminum step-wedge radiographed on the same film. An aluminum step-wedge is now the standard for radiopacity comparisons [20, 21].

Digital X-ray systems have been used in past studies to determine radiopacity [17, 19, 22]. Faster imaging from digital imaging systems is preferred, and can be achieved with less radiation exposure [23]. Choice of imaging method can influence the measured radiopacity values of dental materials. Sabbagh et al. [24] reported a difference of up to 10% in radiopacity values between conventional films and phosphor plate images. Similarly, Akcay et al. [17] found significant differences in radiopacity values of root canal filling materials among different imaging systems, including E-speed film, phosphor plate, and CCD sensor.

The ProRoot MTA, NeoMTA 2 and OrthoMTA materials had radiopacity values exceeding 3 mm Al. Biodentine had an equivalent radiopacity of 2.29 ± 0.21 mm Al. Radiopacity values ranging between 1.5 and 4.1 mm Al have been reported for the radiopacity of Biodentine [13, 25, 26]. Differences between studies may be due to differences in methodology, such as radiography techniques, film-to-focus distance, or density measurement. Kaup et al. [13] used conventional radiography and a densitometer to measure the densities in contrast to other studies. However, digital radiography might provide more standardized results, avoiding any variations in the contrast and density of the materials due to errors inherent in film processing. Another reason for the discrepancy in the radiopacity values in different studies may be due to the storage conditions. Grech et al. [25] immersed the samples in gelatinized Hank’s Balanced Salt Solution (HBSS) for 1 day and 28 days and found radiopacity values of 3.3 Al mm and 4.1 Al mm, respectively.

The radiopacity of MTA Angelus, BioMTA, and Biodentine were compared in another study in which Biodentine showed the lowest radiopacity value of 2.2 mm Al [27]. The radiopacity of Biodentine was found too low for clinical use as its low radiopacity makes it difficult to distinguish it from dental tissues [13]. Similarly, the radiopacity of Biodentine didn’t comply with ISO 6876:2012 requirements in the present study. Low radiopacity has been found to be a disadvantage of this material [28].

ProRoot MTA had an equivalent radiopacity value of 4.32 ± 0.17 mm Al in the present study. Gandolfi et al. [29] reported a radiopacity value of 4.34 ± 0.64 mm Al and Kang et al. [30] reported a value of 4.97 mm Al. Our experiments were consistent with these previous results. However, in a previous study comparing ProRoot MTA and Biodentine, a radiopacity value of 6.40 ± 0.06 mm Al was reported for ProRoot MTA [13]. Although this finding for ProRoot MTA differed from our results, ProRoot MTA was found to be significantly more radiopaque than Biodentine, as reported in a previous study [13]. Our findings revealed lower radiopacity values compared to some studies [31–33]. Khalil et al. [31], in their study, reported radiopacity values of approximately 9 mm Al for ProRoot MTA. However, it is important to highlight that they employed a different methodology compared to our study. The specimens were placed directly on phosphor plates, which may have influenced the results. Additionally, they used a longer exposure time in their study, which differs from both our study and the previous studies [32, 33]. Wang et al. [34] reported a notably higher radiopacity value of 9 mm Al for ProRoot MTA similar to findings of Khalil et al. They employed a unique methodology, using developed film and a digital camera, along with different exposure settings and focus-film distance. Pelepenko et al. used a digital sensor and Torabinejad et al. used film processing and densitometer readings. The radiopacity values obtained were 6.38 mm Al and 7.17 mm Al for ProRoot MTA, respectively [32, 33]. Variations in storage conditions, exposure settings, and imaging techniques may have influenced the lower radiopacity values of the present study compared to the previous studies [31–35].

In the present study, ProRoot MTA showed a significantly higher radiopacity than all other materials. ProRoot MTA, having a higher radiopacity, contains approximately 20% bismuth oxide, while Biodentine contains 5% zirconium oxide [13]. Bismuth oxide has often been used as a radiopacifier in dental materials. However, the cytocompatibility of bismuth oxide is questionable [36]. Bismuth oxide has also been proven to lead to tooth discolouration after MTA placement. Therefore, the development of calcium silicate cements containing other radiopacifiers is an important research topic in dentistry [37]. Zirconium oxide and tantalum oxides are radiopacifiers without discolouration effect compared to bismuth oxide. However, zirconia has a lower radiopacity is lower due to its lower atomic number [38]. Biodentine was found to be the least radiopaque material when compared to OrthoMTA, RetroMTA, and ProCal in a previous study [20]. OrthoMTA was found less radiopaque than RetroMTA. This finding was attributed to higher amounts of Zirconium content of RetroMTA [20]. As reported by Orhan et al. [20], Biodentine showed less radiopacity value compared to OrthoMTA. This is consistent with our findings and can be attributed to the bismuth oxide presence (3.24%) in OrthoMTA. Although Orhan et al. [20] reported radiopacity of 2.56 ± 0.19 mm Al for OrthoMTA, we found higher radiopacity values (3.92 ± 0.09 mm Al). Our experimental set up bears a close resemblance however possible explanation for this inconsistency might be attributed to the irradiation voltage and the X-ray device.

Knowing the radiographic properties of calcium silicate cement materials is useful for root canal treatment. Except for Biodentine, the other three calcium silicate cements had values in accordance with the ISO 6876 and ANSI/ADA 57 standards for root canal materials. Limitations to this study. Include that the powders and liquids were not weighed or measured by volume. Furthermore, in vitro tests do not simulate all intraoral conditions, such as liquid infiltration of such materials. Furthermore, the X-ray phosphor plates’ quality that decreases with reuse [39, 40]. Differences among the findings of studies on radiopacity may be due to differences in the current, voltage, irradiation time, X-ray source, object–source distance, and step wedges.

## Conclusion

ProRoot MTA was the most radiopaque among the 4 tested materials. OrthoMTA and NeoMTA 2 had higher radiopacity values than Biodentine. All materials, except Biodentine, met the radiopacity requirement of ISO 6876.

## Data Availability

The datasets used and analysed during the current study are available from the corresponding author upon reasonable request.

## References

[1] Shah PM, Chong BS, Sidhu SK, Ford TR (1996). Radiopacity of potential root-end filling materials. Oral Surg Oral Med Oral Pathol Oral Radiol Endod.

[2] International Organization for Standardization (2014). ISO 13116. Test Method for determining radio-opacity of materials.

[3] American National Standards Institute/American Dental Association (ANSI/ADA) (2021). Specification number; 57.

[4] International Organization for Standardization (2012). ISO 6876. Dental Root Canal Sealing materials.

[5] Rasimick BJ, Shah RP, Musikant BL, Deutsch AS (2007). Radiopacity of endodontic materials on film and a digital sensor. J Endod.

[6] Torabinejad M, Pitt Ford TR (1996). Root end filling materials: a review. Endod Dent Traumatol.

[7] Palma PJ, Marques JA, Falacho RI (2019). Six-Month Color Stability Assessment of two calcium silicate-based cements used in regenerative endodontic procedures. J Funct Biomater.

[8] Macwan C, Deshpande A (2014). Mineral trioxide aggregate (MTA) in dentistry: a review of literature. J Oral Res Rev.

[9] Kaur M, Singh H, Dhillon JS, Batra M, Saini M (2017). MTA versus Biodentine: review of literature with a comparative analysis. J Clin Diagn Res.

[10] Laurent P, Camps J, De Méo M, Déjou J, About I (2008). Induction of specific cell responses to a ca(3)SiO(5)-based posterior restorative material. Dent Mater.

[11] Kim M, Yang W, Kim H, Ko H (2014). Comparison of the biological properties of ProRoot MTA, OrthoMTA, and endocem MTA cements. J Endod.

[12] Anju PK, Purayil TP, Ginjupalli K, Ballal NV. “Effect of chelating agents on push-out bond strength of NeoMTA Plus to Root Canal Dentin”. Pesquisa Brasileira Em Odontopediatria E Clínica Integrada,2022;(22):1–9.

[13] Kaup M, Schäfer E, Dammaschke T (2015). An in vitro study of different material properties of Biodentine compared to ProRoot MTA. Head Face Med.

[14] Rodríguez-Lozano FJ, Lozano A, López-García S (2022). Biomineralization potential and biological properties of a new tantalum oxide (Ta_2_O_5_)-containing calcium silicate cement. Clin Oral Investig.

[15] Chang SW, Baek SH, Yang HC (2011). Heavy metal analysis of ortho MTA and ProRoot MTA. J Endod.

[16] Gümrü B, Türkaydın D, Sazak Öveçoğlu H (2014). Evaluation of the radiopacity of a MTA based root canal filling material using digital radiography. Clin Exp Health Sci.

[17] Akcay I, Ilhan B, Dundar N (2012). Comparison of conventional and digital radiography systems with regard to radiopacity of root canal filling materials. Int Endod J.

[18] Elíasson ST, Haasken B (1979). Radiopacity of impression materials. Oral Surg Oral Med Oral Pathol.

[19] Beyer-Olsen EM, Orstavik D (1981). Radiopacity of root canal sealers. Oral Surg Oral Med Oral Pathol.

[20] Orhan EO, Irmak Ö, Bal EZ (2021). Radiopacity quantification and spectroscopic characterization of OrthoMTA and RetroMTA. Microsc Res Tech.

[21] Ochoa-Rodríguez VM, Tanomaru-Filho M, Rodrigues EM, Guerreiro-Tanomaru JM, Spin-Neto R, Faria G (2019). Addition of zirconium oxide to Biodentine increases radiopacity and does not alter its physicochemical and biological properties. J Appl Oral Sci.

[22] Ahmetoğlu F (2013). Şimşek N,Keleş A,Ocak M,Altun O. Radiopacity evaluation of three calcium silicate based materials by digital radiography. Clin Dentistry Res.

[23] McDonnell D, Price C. An evaluation of the Sens-A-Ray digital dental imaging system. Dentomaxillofac Radiol. 1993;(22):121–6. 10.1259/dmfr.22.3.8299829 8299829

[24] Sabbagh J, Vreven J, Leloup G (2004). Radiopacity of resin-based materials measured in film radiographs and storage phosphor plate (Digora). Oper Dent.

[25] Grech L, Mallia B, Camilleri J (2013). Investigation of the physical properties of tricalcium silicate cement-based root-end filling materials. Dent Mater.

[26] Corral C, Negregete P, Estay J, Osorio S, Covarrubias C, Olivera Junior OB, Barud H (2018). Radiopacity and chemical assessment of new commercial calcium silicate based cements. Int J Odontostomat.

[27] Coaguila-Llerena H, Ochoa-Rodriguez VM, Castro-Núñez GM, Faria G, Guerreiro-Tanomaru JM, Tanomaru-Filho M (2020). Physicochemical Properties of a Bioceramic Repair Material - BioMTA. Braz Dent J.

[28] Bachoo IK, Seymour D, Brunton P (2013). Clinical case reports using a novel calcium-based cement. Br Dent J.

[29] Gandolfi MG, Siboni F, Prati C (2012). Chemical-physical properties of TheraCal, a novel light-curable MTA-like material for pulp capping. Int Endod J.

[30] Kang TY, Choi JW, Seo KJ, Kim KM, Kwon JS, Physical (2021). Chemical, Mechanical, and Biological Properties of four different Commercial Root-End filling materials: a comparative study. Materials.

[31] Khalil I, Naaman A, Camilleri J (2015). Investigation of a novel mechanically mixed mineral trioxide aggregate (MM-MTA(™)). Int Endod J.

[32] Pelepenko LE, Saavedra F, Antunes TBM (2021). Physicochemical, antimicrobial, and biological properties of White-MTAFlow. Clin Oral Investig.

[33] Torabinejad M, Hong CU, McDonald F, Pitt Ford TR (1995). Physical and chemical properties of a new root-end filling material. J Endod.

[34] Wang CW, Chiang TY, Chang HC, Ding SJ (2014). Physicochemical properties and osteogenic activity of radiopaque calcium silicate-gelatin cements. J Mater Sci Mater Med.

[35] Islam I, Chng HK, Yap AU (2006). Comparison of the physical and mechanical properties of MTA and portland cement. J Endod.

[36] Min KS, Chang HS, Bae JM, Park SH, Hong CU, Kim EC. The induction of heme oxygenase-1 modulates bismuth oxide-induced cytotoxicity in human dental pulp cells. J Endod. 2007 Nov;33(11):1342–6. 10.1016/j.joen.2007.07.012 17963960

[37] Choi Y, Hwang YC, Yu MK, Lee KW, Min KS (2023). Effects of barium titanate on the dielectric constant, radiopacity, and biological properties of tricalcium silicate-based bioceramics. Dent Mater J.

[38] Chen MS, Chen SH, Lai FC (2018). Sintering pmperature-dependence on Radiopacity of Bi_(2–x)_ ZrxO_(3+x/2)_ powders prepared by Sol-Gel process. Mater (Basel).

[39] White S, Pharoah M (2004). Oral radiography principles and interpretation.

[40] Akdeniz BG, Gröndahl H-G, Kose T (2005). Effect of delayed scanning of storage phosphor plates. Oral Surg Oral Med Oral Pathol Oral Radiol Endodontology.

